# On the Influence of Atmospheric Pressure on the Circulation of the Blood: Being a Reply to the Observations of Dr. Arnott on That Subject

**Published:** 1827-08

**Authors:** 


### PHYSIOLOGY.

### *On the Influence of Atmospheric Pressure on the Circulation of the Blood: being a Reply to the Observations of* Dr. Arnott *on that subject.* By —— Carson, M.D. &c. &c.*

In the Number of the London Medical Journal for June 1827, there appears an extract from a work on Physics, lately published by Dr. Arnott. As this selection from Dr. Arnott's work appears to be the voluntary act of the editor of the Journal, and is introduced with an observation expressive of his concurrence with the argument maintained in it, he will be disposed, I am sure, to give an early place to the following remarks.

In the extract it is stated that Dr. Carson and Dr. Barry had wasted a great deal of ingenuity in attempting to prove that the blood is returned to the heart by atmospherical pressure.

The errors into which Dr. Barry and myself have fallen, Dr. Arnott very delicately and with kind indulgence attributes not to our want of talents, but to a deficiency in our education. As it appears that an alarming number of other medical gentlemen, also not deficient in talents, had adopted the same sentiment, Dr. Arnott takes this opportunity of making some very modest, and I have no doubt very useful, observations on the deficiency of the education generally of medical students. The branch of education of which Dr. Arnott laments the deficiency, is evidently the mathematical sciences. For high attainments in these sciences, I have ever felt the greatest veneration, not unaccompanied with a wish and some efforts that these should be my own; and I am ready to allow that, in the course of my physiological inquiries respecting the motion of the blood and respiration, I have had many occasions to lament that these efforts were not more successful. But their want of success is far from being acknowledged in the treatment of the subject discussed in this extract.

It has been observed that great progress may be made by some men, even in the higher branches of mathematics, with-

* In the MS. this paper is merely entitled "Dr. Carson's Reply to Dr. Arnott," but we have thought it necessary to substitute the above, as more explicit.—E.

out much expenditure of thinking. So long as these men are allowed to adhere to their *formulæ*, they will advance with great correctness and rapidity, but, if left a moment without their accustomed guide, they soon come to a stand, or deviate into bewildering errors. They are like a waggon in a rail-road: as long as the wheels of the waggon roll within the iron grooves of the rail-way, the machine proceeds with correctness, safety, and often with wonderful rapidity; but, if the wheels slip out of their defence, the machine either stops entirely, or, if it moves, is soon overthrown, scattering its diversified contents in ruinous disorder along the plain. Dr. Arnott would appear to afford an illustration of the correctness of this observation. But let it be understood that I am drawing this conclusion from the extract alone which the editor has published, as I have not seen the rest of that gentleman's work, which may in other articles be deserving of the praise bestowed upon it. But to proceed to the Doctor's argument. Any person, the learned Doctor observes, who understands the nature of a domestic pump, must be satisfied that blood cannot be raised by suction through pliant vessels like the veins, as the sides of the vessel will be pressed together by the atmosphere, or will collapse, and thus an end will be put to suction.

When engaged in the inquiry into the causes of the motion of the blood, and looking out for objections which might be urged against the doctrine, that I might prevent them by an anticipated reply, this occurred as one that would most readily be made, and one to which careless and superficial reasoners would appear plausible and of great weight. The objection was nevertheless repeatedly urged, and in particular by Dr. Wilson Philip, I think, in the Medical and Chirurgical Transactions. To Dr. Philip I took occasion to repeat my reply in a Treatise on the Motion of the Blood in the Head, published in the Edinburgh Medical and Surgical Journal, and was in hopes that this reply would have prevented the renewal of the objection. In this, however, I have been disappointed. As this reply has either been unobserved or deemed defective by the learned author of the Treatise on Physics and the editor of the London Journal, I am compelled to resume the subject; and, as I hope it will be for the last time, will go into it to greater extent.

If the fluid to be raised by a pump be of a greater specific gravity than the fluid through which it is to be raised, it is necessary that the stem of the pump be so far incompressible as to resist a greater pressure than that supplied by a column of fluid of the height of the pump, and of a weight measurable by the difference of the specific gravity of the fluid to be raised and of that through which it is to be raised. In the case of raising water through the medium of air, such a pump is necessary. Any attempt to raise water in this way through the atmosphere in easily compressible tubes will necessarily fail, in consequence of the collapsing of the tube. But the case is very different if the fluid to be raised be of the same specific gravity with the fluid through which it is to be raised: for instance, if water is to be raised through water, a very limber and pliant tube will be sufficient for the purpose, as the pressure, in consequence of the equality of the gravity of the fluids, must be as great upon the internal as upon the external surface of the tube. According as the fluids to be raised exceed or approach to the fluids through which they are to be raised in specific gravity, in the same proportion will the stem of a pump of any given height require to be more or less unyielding. A tube with weaker sides will be sufficient to raise oil through air, than would be required to raise water to a given height through the same medium; and a firmer tube will be necessary for raising mercury through air, than what would be required to raise the same metal through water to a given height. It is not simply, therefore, the removal of the atmospherical pressure from the surface of the fluid to be raised which regulates the degree of pressure sustained by the external surface of the pump, but it is that in conjunction with the difference of the gravity of the fluids to be raised, and that of the medium through which they are to be raised.

These facts, which (simple as they appear) are of the greatest importance in our present inquiry, may all be proved by an easy experimental process. Take a very limber and pliant onion-stalk, of a foot, say, in length, and insert one end of it into water, and suck through the other end; the sides of the stalk will collapse, and no water will reach the mouth. But, if the stalk be sunk to the top in water, and an attempt be made in that state to suck from the upper end, the water will flow readily into the mouth, and the sides of the tube will manifest no tendency to collapse. The same thing will occur in attempting to draw air through air, oil through the medium of oil, or mercury through that of mercury. In case, in these last instances, the suction be made so that the fluid should pass with great rapidity through the tube, the sides of the tube may yield inwards to a certain extent, in consequence of the resistance given to the passage of the fluid through the tube by the friction between the sides of the tube and the current. It plainly follows that the force or the extent of the removal of atmospherical pressure required to raise fluids through tubes will be in proportion to the difference between the specific gravity of the fluid to be raised and that of the fluid through which it is to be raised, the height being the same. Greater suction will be required to raise mercury to the height of an inch through air, than would be required to raise water to the same height in the same medium. Hence it follows that less diminution of resistance will be sufficient to return the blood to the heart through the veins of fishes, and the fœtus in utero, which are constantly immerged in a fluid nearly of the same specific gravity with the blood, than in those of the mammalia after birth. The power derived from the simple relaxation of the circular fibres of the heart, aided by the elasticity perhaps of those fibres, will be quite sufficient to secure the return of the blood to the heart in fishes and in the fœtus in utero. In animals so situated, any further aid from lungs would be superfluous, and is therefore not supplied. For the same reason, no obstruction is to be apprehended to the return of the blood from the collapsing of the sides of the vein. One would infer from this doctrine, that fishes are little subject to inflammation. A great deal of the advantage derived from sea-bathing might be accounted for in this principle, and bathing, or more effectually perhaps the diving bell, might be extended with advantage to many diseases, in which the freedom of the circulation was affected. In the mammalia, after birth, the veins are placed in a situation not altogether the same with that of those vessels in fishes and in the fœtus in utero; though, even in the former, these vessels may be said to be immersed in a fluid, or in a substance charged with a fluid, of the same specific gravity with that of the fluid which it is their office to transmit.

The veins of the mammalia can neither be said to be in the situation of these vessels in fishes or in the fœtus in utero; nor are they in the state of vessels immersed in air, or in a fluid of a specific gravity very different from that of the fluid they contain. They are in a middle state, but one which approaches nearer to that of the same vessels in fishes, than that of a tube solely surrounded by air. The heart, aided probably by the arteries, transmits blood to all the extreme arteries; by which the most extreme parts of the system are pervaded. By the rapidity with which these transmissions are made, the system may be said to be kept constantly full, although its sides are compressible and surrounded by air. The animal system may be compared to a vessel of fixed dimensions, of the same size and shape, containing solid materials of various dimensions and forms, frequently hollow, while all the cavities and interstices are filled with blood, watery fluids, or air under a considerable degree of compression, excepting the air in the bronchi, which may be considered as exterior to the vessel. A vein traversing a fabric of this description is nearly, though not altogether, in the same situation with a hollow tube fully immersed in any homogeneous fluid. The sides of the vein, when blood was attempted to be transmitted through it, might have some tendency to collapse. But this tendency is counteracted by a variety of causes. The vein is not bare, but has a space assigned to it fitted to its bulk with its ordinary charge of blood. This space it must always occupy, or leave a space to be occupied by some other substance, more or less fixed. It is connected throughout its course with other substances, which, by collapsing, it must draw from other connexions. The arched structure, particularly in the capillaries and smaller veins, where the curvature of the arches is great, and which often run upon surfaces in contact with air, enables it to resist considerable external force. On the supposition that blood is returned to the heart by atmospherical pressure, a considerable force must be required to remove the requisite degree of pressure from the end of the tube through which the blood is to be discharged into the heart; but by no means so great a force as would be required to raise water to an equal height through air. In the case of the mammalia after birth, simple relaxation of the circular fibres of the heart, aided, as that dilating relaxation may be, by an elastic structure, might not be sufficient, a further aid is afforded by the elasticity of the lungs.

But, says Dr. Arnott, there are certain phenomena which show that if it were possible to return the blood by suction to the heart in this way: this, however, is not required; and in support of his assertion he adduces the operation of bleeding. If the veins of the arm are compressed by a ligature, the veins beyond the ligature will soon be seen to swell, and, if the vein be opened beyond the ligature, blood will flow with great force. This force cannot, he justly observes, be occasioned by suction. If Dr. Arnott had perused with any attention my Treatise on the Motion of the Blood, he would not have, I think, stated this objection. I do not deny that the force of the heart and arteries may be sufficient to move the blood in certain circumstances through its whole course, but maintain that this is not the force employed for that purpose by nature. Before the action or impetus of the heart and arteries can effect the blood in the veins, it is necessary that the veins be distended to that condition in which they possess the property of rigid tubes. Now, in the case adduced by Dr. Arnott, in consequence of the veins being rendered impermeable, and blood being constantly poured into them by the arteries, they soon become distended to their utmost capacity, and in that situation act like rigid tubes. A quantity of blood being thrown into them in this situation at one end, an equal quantity must be discharged by the same force from the other end. In the ordinary circulation, the veins are by no means dilated to this extent. Therefore, however powerful the action of the heart and arteries may be, the blood cannot be transmitted through the veins by this action. Allow me to ask, in my turn, one question of Dr. Arnott: When a vein is opened in the arm or leg, without the veins above the opening being compressed by ligatures, why is not blood discharged from these openings? The blood certainly, on the supposition of its being propelled by a force *a tergo*, would find its way rather out of the opening, than proceed in the vein, carrying before it a column of blood one or two feet in height.

It is not a little surprising, and I think indicative of bad generalship, that, in attempting to refute the doctrine that the blood is returned to the heart by atmospherical pressure, Dr. Arnott should have reference to the explication of phenomena. It is a matter of surprise to many physiologists of the present day, and I doubt not will be so to many more in another age, that such reluctance has been shown to abandon doctrines with which the greatest ingenuity is incapable of reconciling the most common occurrences, and such hesitation in embracing an hypothesis by which all the phenomena referable to it admit of an easy and satisfactory explication. The single fact that little or no blood flows from a wound made in a vein in the leg or arm, is sufficient to overturn on this point the doctrine of Harvey, which maintains that blood in the veins is returned to the heart by the projectile power of the left ventricle alone, and which subsequent physiologists have not improved by enlisting the vibrations of the arteries in aid of the power of the heart. Dr. Arnott denies that Dr. Barry's experiment with cupping-glasses, by which that physician stopped the progress of poison introduced into a wound, is any proof of the influence of suction in the motion of the blood. It appears, however, to me, that if a substance be stopped in its progress by removing from it a share of atmospherical pressure, that atmospherical pressure must have some part in the process allowed to be made by that substance, when no share of that pressure is withdrawn. Dr. Arnott seems displeased that Dr. Barry should attempt to erect a system of practice upon the doctrine of the blood being returned to the heart by atmospherical pressure. I trust, however, that Dr. Barry will not be deterred from his pursuits by menaces, I may call them, of this nature. There is not an object, in my opinion, more worthy of the talents of Dr. Barry, nor one by pursuing which he can render greater services to mankind, than by a careful application of the doctrines which, with some modifications, he has espoused in the complicated phenomena of disease, and of subjecting the medical practice to the same, as I think, infallible test.

What obscurity still hangs over the doctrine of inflammation? How disappointing, how contradictory, and frequently how pernicious, the treatment of it! If the hypothesis that the blood is returned to the heart by atmospherical pressure be true,—and stronger arguments must be produced than any which have hitherto been urged before I can have any doubt of its truth,—the practice hitherto employed in all diseases depending on a derangement of the circulating fluids must have been either empirical, (by which I mean, derived solely from experience,) or founded on an erroneous hypothesis.

*Liverpool; 7th July*, 1827.

